# Fat distribution, inflammatory mechanisms, and cardiovascular disease risk: mediation analysis based on the Framingham risk score

**DOI:** 10.1186/s12872-025-05135-3

**Published:** 2025-09-26

**Authors:** Zhuangzhuang Chen, Jun Li, Hao Rao, Jia Zhang, Zhili Xiao, Wei Sun, Mingzhong Xiao

**Affiliations:** 1 First Clinical College, Hubei University of Chinese Medicine, Wuhan, 430065 China; 2 School of Traditional Chinese Medicine, Hubei University of Chinese Medicine, Wuhan, 430065 China; 3 Institute of Liver Diseases, Hubei Key Laboratory of the theory and application research of liver and kidney in traditional Chinese medicine, Hubei Provincial Hospital of Traditional Chinese Medicine, Wuhan, 430061 China; 4 Affiliated Hospital of Hubei University of Chinese Medicine, Wuhan, 430061 China; 5 Hubei Province Academy of Traditional Chinese Medicine, Wuhan, 430074 China

**Keywords:** Fat distribution, Cardiovascular disease, Framingham risk score, Inflammatory markers, C-reactive protein, Mediation analysis, NHANES database

## Abstract

**Objective:**

To examine the association between fat distribution and 10-year cardiovascular disease (CVD) risk, and to evaluate the mediating roles of inflammatory markers in this relationship.

**Methods:**

Data were obtained from the 2017–2018 cycle of the National Health and Nutrition Examination Survey (NHANES), including 4,741 participants aged ≥ 30 years. Fat distribution was assessed using body mass index (BMI), waist circumference (WC), and regional fat percentages (upper limbs, lower limbs, trunk, and total body) measured via dual-energy X-ray absorptiometry (DXA). The Framingham Risk Score (FRS) was used to estimate 10-year cardiovascular disease (CVD) risk. Multivariable linear regression models were applied to evaluate the associations between fat distribution and FRS. In addition, mediation analysis was performed to assess the indirect effects of inflammatory markers—including C-reactive protein (CRP), white blood cell count (WBC), and neutrophil-to-lymphocyte ratio (NLR)—on the relationship between fat distribution and FRS. All analyses were stratified by sex.

**Results:**

In men, the total fat percentage showed the strongest positive correlation with the Framingham Risk Score (FRS) (β = 0.08, 95% CI: 0.07–0.10), followed by the trunk fat percentage (β = 0.09, 95% CI: 0.08–0.11) and BMI (β = 0.07, 95% CI: 0.06–0.08). C-reactive protein (CRP) exhibited a significant mediating effect in the relationship between total fat and FRS (indirect effect = 0.019, 95% CI: 0.015–0.025), with a mediation proportion of 23.1%.

In women, total fat percentage demonstrated a significant "U-shaped" nonlinear relationship with FRS (P_nonlinear = 0.046), with risk increasing sharply when body fat exceeded approximately 35%. In the fully adjusted model, the three major fat distribution indicators influencing FRS were total fat percentage (β= 0.11, 95% CI: 0.07–0.14), trunk fat percentage (β = 0.11, 95% CI: 0.07–0.14), and BMI (β = 0.09, 95% CI: 0.07–0.11). The mediating effect of CRP was the most significant (indirect effect = 0.041, 95% CI: 0.031–0.054), with a mediation proportion of 38.1%.

**Conclusion:**

Different fat distribution patterns exert varying influences on long-term cardiovascular risk, with total body fat percentage showing the strongest association with increased risk. Inflammatory responses may serve as key biological mediators linking fat distribution to cardiovascular disease (CVD), highlighting the importance of monitoring and managing inflammation to reduce CVD risk.

## Introduction

Cardiovascular disease (CVD) comprises a group of common chronic non-communicable disorders primarily driven by atherosclerosis. These conditions affect the heart and vascular system and include coronary artery disease, stroke, and hypertension [1]. Atherosclerosis is now widely recognized as a progressive, systemic pathology fueled by chronic low-grade inflammation [2]. According to the World Health Organization (WHO), CVD remains the leading cause of mortality worldwide, accounting for approximately 17.8 million deaths in 2019—about one-third of all global deaths [3, 4]. The burden is particularly pronounced in low- and middle-income countries, where limited healthcare access and suboptimal risk factor control contribute to disproportionately high mortality [5]. Notably, CVD has shown a disturbing trend toward earlier onset, with a rising prevalence among younger adults [6]. These epidemiological patterns underscore the urgent need to identify modifiable risk factors and elucidate underlying biological mechanisms to facilitate early detection and precision prevention strategies.

Abnormal fat accumulation is a well-established risk factor for cardiovascular disease (CVD). However, recent evidence indicates that the pattern of fat distribution, rather than total adiposity, more accurately reflects an individual’s metabolic risk and cardiovascular burden. Traditional metrics, such as body mass index (BMI), are widely utilized but fail to distinguish between fat and lean mass, and cannot capture the heterogeneous health impacts of regional fat deposition [7, 8]. Central adiposity—particularly abdominal visceral and trunk fat—has been identified as a key driver of preclinical CVD states, including metabolic dysregulation, insulin resistance, and atherosclerosis, due to its elevated lipolytic activity and pro-inflammatory secretory profile [9]. The Chinese Visceral Adiposity Index (CVAI), for instance, has demonstrated superior predictive value for metabolic syndrome, even among individuals with normal BMI, further underscoring the significance of fat distribution over total fat mass in identifying cardiometabolic risk [10]. Emerging studies have also shown that specific fat depots, such as epicardial adipose tissue, play an active role in atherogenesis through the release of extracellular vesicles that mediate crosstalk among adipose, vascular, and immune systems [11]. Notably, each one–standard deviation increase in abdominal fat has been associated with a significant elevation in CVD event risk. This heightened risk is partly mediated by the secretion of inflammatory factors such as C-reactive protein (CRP) and tumor necrosis factor-alpha (TNF-α), which contribute to plaque formation and instability [12–15].

In contrast, lower limb fat is thought to exert a “metabolic buffering” effect. It is positively associated with adiponectin levels, enhances insulin sensitivity, regulates lipid metabolism, and is linked to a reduced risk of cardiovascular events—an effect that appears particularly pronounced in women [16, 17]. This regional heterogeneity in fat function may be attributed to differences in lipase activity, macrophage polarization, and hormonal regulation [18–20]. In comparison, the role of upper limb fat remains inconclusive. Some studies suggest a U-shaped association between upper limb fat and levels of systemic inflammation or mortality, indicating that environmental exposures—such as smoking—may modulate its metabolic consequences [21].

However, existing literature has primarily focused on waist circumference and visceral fat measured by abdominal computed tomography (CT), while systematic comparisons of regional fat distribution—including upper limbs, lower limbs, and trunk fat percentages assessed via dual-energy X-ray absorptiometry (DXA)—remain limited [22]. Notably, a growing body of evidence highlights chronic low-grade inflammation as a key biological mediator linking fat distribution to cardiovascular disease (CVD) risk. Adipose tissue, particularly visceral fat, secretes a range of pro-inflammatory factors—such as C-reactive protein (CRP), white blood cell count (WBC), and the neutrophil-to-lymphocyte ratio (NLR)—that may promote persistent systemic inflammation. This inflammatory state contributes to endothelial dysfunction, atherosclerosis, and the progression of CVD events [23, 24]. Nevertheless, most prior studies have focused on individual inflammatory markers, lacking a comprehensive evaluation of multiple biomarkers and a quantitative analysis of their mediating roles in the fat–CVD pathway [25]. In contrast to traditional cardiovascular risk factors—such as ABO blood group—which have demonstrated inconsistent associations with thrombotic burden and ischemic events in acute coronary syndromes, fat distribution and its inflammatory sequelae represent modifiable targets with more clearly defined mechanistic links to chronic CVD development [26]. Furthermore, recent studies have emphasized the prognostic value of inflammation-related indices, such as the HALP score, in acute cardiovascular settings, suggesting that systemic immune-nutritional status may significantly influence cardiovascular outcomes [27].

Building on these gaps, the present study draws on nationally representative data from the 2017–2018 National Health and Nutrition Examination Survey (NHANES) to systematically investigate the associations between fat distribution across multiple anatomical regions—including upper limbs, lower limbs, trunk, and total body fat percentage—and the 10-year Framingham Risk Score (FRS). For the first time, we integrate multiple inflammatory markers to evaluate their mediating roles in the pathway linking fat distribution to cardiovascular disease (CVD) risk, with the goal of constructing a mechanistic model of the fat–inflammation–CVD axis. This framework is designed to offer both mechanistic insights and practical guidance for individualized CVD risk assessment and targeted intervention.

Based on this rationale, the present study formulates the following hypotheses:

- *H1*: Fat percentages in different anatomical regions are significantly and positively associated with 10-year cardiovascular disease (CVD) risk, as estimated by the Framingham Risk Score (FRS).
- *H2*: Multiple inflammatory markers—such as C-reactive protein (CRP) and white blood cell count (WBC)—significantly mediate these associations, with potential sex-specific differences in both biological pathways and effect magnitudes.

The study is designed to systematically test these hypotheses and further investigate the sex-specific roles of regional fat distribution in the pathophysiology of cardiovascular risk.

## Materials and methods

### Data source and study population

This study was based on data from the 2017–2018 cycle of the National Health and Nutrition Examination Survey (NHANES), a program conducted by the U.S. Centers for Disease Control and Prevention (CDC) using a multistage, stratified sampling design. The survey encompasses demographic information, health behaviors, physical examinations, and laboratory assessments, offering high population representativeness. A total of 4,741 adult participants aged 30 years and older were included in the final analysis.

**Fig. 1:**
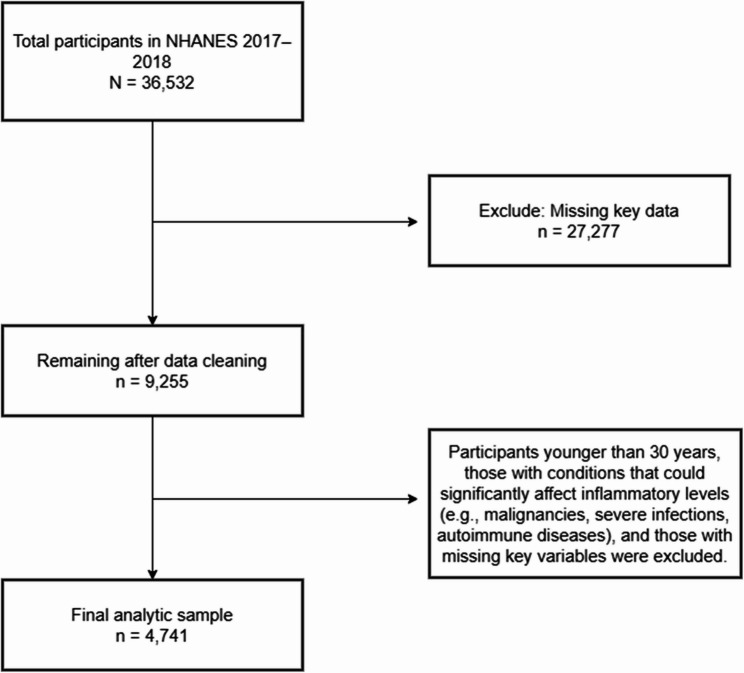
Inclusion and exclusion criteria for the analytic sample based on NHANES 2017–2018

Inclusion criteria were: age ≥ 30 years and availability of complete data required to calculate the Framingham Risk Score (FRS), including age, sex, systolic blood pressure, total cholesterol, high-density lipoprotein cholesterol (HDL-C), smoking status, diabetes status, as well as complete measures of body composition (BMI, waist circumference, and DXA-derived fat percentages for upper limbs, lower limbs, trunk, and total body). Participants were excluded if they were under 30 years of age, had major illnesses affecting inflammatory status (e.g., malignancies, severe infections, autoimmune diseases), or had missing data on key variables, as outlined in Fig. 1.

### Variable definitions and measurements

The primary exposure variables in this study were indicators of fat distribution, including body mass index (BMI; calculated as weight in kilograms divided by height in meters squared, kg/m²), waist circumference (WC; measured at the midpoint between the lowest rib and the iliac crest in the standing position), and regional fat percentages—specifically in the upper limbs, lower limbs, trunk, and total body—assessed using dual-energy X-ray absorptiometry (DXA).The mediating variables were inflammatory biomarkers, including high-sensitivity C-reactive protein (CRP), total white blood cell count (WBC), neutrophil count, lymphocyte count, and platelet count, all of which were measured using standardized hematology analyzers. The neutrophil-to-lymphocyte ratio (NLR) was calculated as the ratio of absolute neutrophil count to lymphocyte count.

The primary outcome variable in this study was the Framingham Risk Score (FRS), a widely accepted and clinically validated tool for estimating 10-year cardiovascular disease (CVD) risk. FRS incorporates traditional risk factors—including age, sex, systolic blood pressure, total cholesterol, high-density lipoprotein (HDL) cholesterol, smoking status, diabetes status, and antihypertensive medication use—and is endorsed by major clinical guidelines such as those from the American Heart Association (AHA) [28]. FRS was selected for its robust predictive performance (e.g., area under the curve [AUC] ≈ 0.75 in Asian populations), ease of use, and suitability across diverse clinical and research settings [29]. Unlike more complex algorithms, it relies solely on routinely collected clinical variables, making it particularly practical for large-scale population screening and individualized CVD risk assessment.To minimize confounding, all analyses were adjusted for key demographic variables (age, sex, race/ethnicity, education level, and family income) as well as lifestyle factors, including smoking and alcohol consumption.

1 $$\begin{aligned} \:\varvec{F}\varvec{R}\varvec{S}\:=&\:\varvec{\upbeta\:}_{0}\:+\:\varvec{\upbeta\:}_{1}\:\mathbf{l}\mathbf{n}\left(\mathbf{A}\mathbf{g}\mathbf{e}\right)\:+\:\varvec{\upbeta\:}_{2}\:\mathbf{l}\mathbf{n}\left(\mathbf{T}\mathbf{o}\mathbf{t}\mathbf{a}\mathbf{l}\:\mathbf{C}\mathbf{h}\mathbf{o}\mathbf{l}\right)\:\\&+\:\varvec{\upbeta\:}_{3}\:\mathbf{l}\mathbf{n}\left(\mathbf{H}\mathbf{D}\mathbf{L}\right)\:+\:\varvec{\upbeta\:}_{4}\:\mathbf{l}\mathbf{n}\left(\mathbf{S}\mathbf{B}\mathbf{P}\right)\:+\:\varvec{\upbeta\:}_{5}\:\mathbf{S}\mathbf{m}\mathbf{o}\mathbf{k}\mathbf{i}\mathbf{n}\mathbf{g}\:\\&+\:\varvec{\upbeta\:}_{6}\:\mathbf{D}\mathbf{i}\mathbf{a}\mathbf{b}\mathbf{e}\mathbf{t}\mathbf{e}\mathbf{s}\:+\:\varvec{\upbeta\:}_{7}\:\mathbf{T}\mathbf{r}\mathbf{e}\mathbf{a}\mathbf{t}\mathbf{m}\mathbf{e}\mathbf{n}\mathbf{t}\end{aligned}$$

### Statistical analysis

Descriptive statistics were first conducted for all variables. Continuous variables were presented as means ± standard deviations (Mean ± SD), while categorical variables were summarized using frequencies and percentages (n, %). Multivariable linear regression models were then constructed to evaluate the associations between fat distribution indicators—including BMI, waist circumference, and DXA-measured fat percentages of the upper limbs, lower limbs, trunk, and total body—and the Framingham Risk Score (FRS), with adjustments for demographic and lifestyle-related covariates.To explore potential non-linear associations between total body fat percentage and FRS, restricted cubic spline (RCS) models were employed, and sex-specific curves were plotted to visualize the trend in cardiovascular risk across varying levels of fat accumulation.

For mediation analysis, the mediation package in R was utilized to assess the indirect effects of multiple inflammatory markers—including CRP, WBC, neutrophil count, lymphocyte count, platelet count, and NLR—on the association between fat distribution and FRS, stratified by sex. A bootstrapping procedure with 5,000 resamples was performed to estimate the 95% confidence intervals (CI) for the indirect effects and to evaluate the statistical significance of the mediation pathways.

## Results

Table 1 presents the baseline characteristics of the study population, comprising a total of 4,741 participants—2,295 men and 2,446 women. Compared to men, women tended to be younger, with a significantly higher proportion aged 30–59 years (55.7% vs. 51.7%, *p* = 0.006). A greater proportion of women reported a history of prior marriage (33.0% vs. 20.6%, *p* < 0.001). Marked sex differences were also observed in smoking and alcohol consumption behaviors: current smoking (21.7% vs. 14.6%, *p* < 0.001) and frequent drinking (41.4% vs. 37.0%, *p* < 0.001) were both more prevalent in men. Moreover, men had a significantly higher prevalence of diabetes compared to women (24.2% vs. 19.5%, *p* < 0.001).

**Table 1:** Demographic and clinical characteristics of the study population (*N* = 4,741)

Characteristic	Gender		*p*-value
	Male, *N* = 2,295^1^	Female, *N* = 2,446^1^	
Age			0.006^2^
≥ 30, ˂59	1,165 (51.7%)	1,336 (55.7%)	
≥ 60	1,089 (48.3%)	1,061 (44.3%)	
Race/Hispanic origin			0.144^2^
Mexican American	278 (12.1%)	313 (12.8%)	
Non-Hispanic White	855 (37.3%)	834 (34.1%)	
Non-Hispanic Black	525 (22.9%)	581 (23.8%)	
Other Hispanic	193 (8.4%)	241 (9.9%)	
Other Race - Including Multi-Racial	444 (19.3%)	477 (19.5%)	
Education level - Adults 20+			0.146^2^
Low education	510 (22.2%)	495 (20.2%)	
Middle education	1,209 (52.7%)	1,352 (55.3%)	
High education	576 (25.1%)	599 (24.5%)	
Marital status			< 0.001^2^
Married	1,560 (68.0%)	1,364 (55.8%)	
Previously married	473 (20.6%)	807 (33.0%)	
Unmarried	262 (11.4%)	275 (11.2%)	
Ratio of family income to poverty			0.018^2^
Low income	496 (21.6%)	609 (24.9%)	
Middle income	720 (31.4%)	707 (28.9%)	
High income	1,079 (47.0%)	1,130 (46.2%)	
Smoking			< 0.001^2^
Current smoker	497 (21.7%)	358 (14.6%)	
Former smoker	782 (34.1%)	450 (18.4%)	
Never smoker	1,016 (44.3%)	1,638 (67.0%)	
Alcohol drinks			< 0.001^2^
Non-drinker	123 (5.4%)	316 (12.9%)	
Occasional drinker	1,222 (53.2%)	1,226 (50.1%)	
Frequent drinker	950 (41.4%)	904 (37.0%)	
Systolic. Blood pres mm Hg	127 ± 21	126 ± 22	0.209^3^
Diastolic. Blood pres mm Hg	74 ± 13	71 ± 13	< 0.001^3^
Taking prescription for hypertension			0.774^2^
Yes	927 (40.4%)	978 (40.0%)	
No	1,368 (59.6%)	1,468 (60.0%)	
Doctor told you have diabetes			< 0.001^2^
Yes	555 (24.2%)	477 (19.5%)	
No	1,740 (75.8%)	1,969 (80.5%)	
Body Mass Index (kg/m**2)	30 ± 6	30 ± 8	< 0.001^3^
Waist Circumference (cm)	104 ± 15	100 ± 16	< 0.001^3^
Upper limb fat content percentage	11 ± 13	17 ± 21	< 0.001^3^
Lower limb fat percentage	11 ± 14	18 ± 21	< 0.001^3^
Trunk Percent Fat	31.9 ± 4.7	35.0 ± 4.9	< 0.001^3^
Total Percent Fat	32.1 ± 4.4	36.2 ± 4.6	< 0.001^3^
Framingham Risk Score (FRS)	7.1 ± 2.8	7.7 ± 6.4	< 0.001^3^

^1^*n* (%); Mean ± SD

^2^Pearson’s Chi-squared test

^3^Welch Two Sample t-test

In terms of fat distribution, women exhibited significantly higher total body fat percentage (36.2% vs. 32.1%, *p* < 0.001), as well as higher fat percentages across all measured regions (upper limbs, lower limbs, and trunk). Notably, women also had higher Framingham Risk Scores (FRS) than men (7.7 vs. 7.1, *p* < 0.001), suggesting a greater estimated 10-year CVD risk. However, men displayed higher diastolic blood pressure than women (74 mmHg vs. 71 mmHg, *p* < 0.001). No significant sex differences were found in systolic blood pressure, use of antihypertensive medication, or racial/ethnic distribution.

Table 2. Association Between Fat Distribution and Framingham Risk Score (FRS).Among male participants, linear regression analyses revealed that total body fat percentage was the strongest predictor of the Framingham Risk Score (FRS) across all fat distribution indicators. In the unadjusted model, each 1-unit increase in total fat percentage was associated with a significant increase of 0.26 in FRS (β = 0.26, 95% CI: 0.23–0.28, *p* < 0.001). After adjusting for sociodemographic factors (age, race/ethnicity, education level, etc.), the effect was attenuated but remained statistically significant (β = 0.09, 95% CI: 0.07–0.10, *p* < 0.001). In the fully adjusted model—including both sociodemographic and clinical covariates such as smoking, alcohol intake, and blood pressure—the association between total fat percentage and FRS persisted (β = 0.08, 95% CI: 0.07–0.10, *p* < 0.001).

**Table 2:** *Sex-Stratified Linear Regression Analysis of Fat Distribution Indicators and Framingham Risk Score (FRS) Across Three Adjustment Models*

Characteristic	Model 1			Model 2			Model 3		
	Beta	95% CI^1^	*p*-value	Beta	95% CI^1^	p-value	Beta	95% CI^1^	*p*-value
Male									
Body Mass Index (kg/m**2)	0.02	0.00, 0.04	0.071	0.06	0.05, 0.08	<0.001	0.07	0.06, 0.08	<0.001
Waist Circumference (cm)	0.03	0.03, 0.04	<0.001	0.03	0.02, 0.04	<0.001	0.03	0.03, 0.04	<0.001
Upper limb fat content percentage	-0.11	-0.12, -0.10	<0.001	-0.01	-0.02, -0.01	<0.001	-0.01	-0.02, 0.00	<0.001
Lower limb fat percentage	-0.10	-0.11, -0.10	<0.001	-0.02	-0.02, -0.01	<0.001	-0.01	-0.02, -0.01	<0.001
Trunk Percent Fat	0.22	0.19, 0.24	<0.001	0.09	0.07, 0.11	<0.001	0.09	0.08, 0.11	<0.001
Total Percent Fat	0.26	0.23, 0.28	<0.001	0.09	0.07, 0.10	<0.001	0.08	0.07, 0.10	<0.001
Female									
Body Mass Index (kg/m**2)	0.08	0.05, 0.11	<0.001	0.10	0.08, 0.12	<0.001	0.09	0.07, 0.11	<0.001
Waist Circumference (cm)	0.08	0.06, 0.09	<0.001	0.06	0.05, 0.07	<0.001	0.05	0.04, 0.06	<0.001
Upper limb fat content percentage	-0.11	-0.13, -0.10	<0.001	0.02	0.01, 0.03	<0.001	0.02	0.01, 0.03	<0.001
Lower limb fat percentage	-0.12	-0.14, -0.11	<0.001	0.01	0.00, 0.02	0.005	0.02	0.01, 0.03	<0.001
Trunk Percent Fat	-0.04	-0.09, 0.02	0.169	0.12	0.09, 0.16	<0.001	0.11	0.07, 0.14	<0.001
Total Percent Fat	-0.16	-0.21, -0.10	<0.001	0.12	0.09, 0.16	<0.001	0.11	0.07, 0.14	<0.001

^1^*CI* Confidence Interval

Model 1 no covariates were adjusted Model 2 adjusted for Age in years at screening, Race/Hispanic origin, Education level - Adults 20+, Marital status, and Ratio of family income to poverty Model 3 adjusted for Age in years at screening, Race/Hispanic origin, Education level - Adults 20+, Marital status, Ratio of family income to poverty, Smoking, Alcohol drinks, Systolic. Blood pres mm Hg, and Diastolic. Blood pres mm Hg

Waist circumference and BMI also showed significant positive associations with FRS, although their effect sizes were smaller than that of total fat percentage. In the fully adjusted model, a 1 cm increase in waist circumference was associated with a 0.03-point increase in FRS (β = 0.03, 95% CI: 0.03–0.04, *p* < 0.001), and each 1-unit increase in BMI corresponded to a 0.07-point increase in FRS (β = 0.07, 95% CI: 0.06–0.08, *p* < 0.001). These findings underscore that total fat percentage, waist circumference, and BMI are key predictors of cardiovascular risk in men, with total fat being the most prominent factor.

In female participants, total fat percentage also emerged as the most significant predictor of FRS. Interestingly, in the unadjusted model, total fat percentage was negatively associated with FRS (β = − 0.16, 95% CI: − 0.21 to − 0.10, *p* < 0.001). However, after adjustment for demographic variables such as age, race, education, marital status, and income, the direction of association reversed, becoming significantly positive (β = 0.12, 95% CI: 0.09–0.16, *p* < 0.001). This positive association remained robust after further adjustment for clinical covariates, including smoking, alcohol consumption, systolic, and diastolic blood pressure (β = 0.11, 95% CI: 0.07–0.14, *p* < 0.001).

**Fig. 2:**
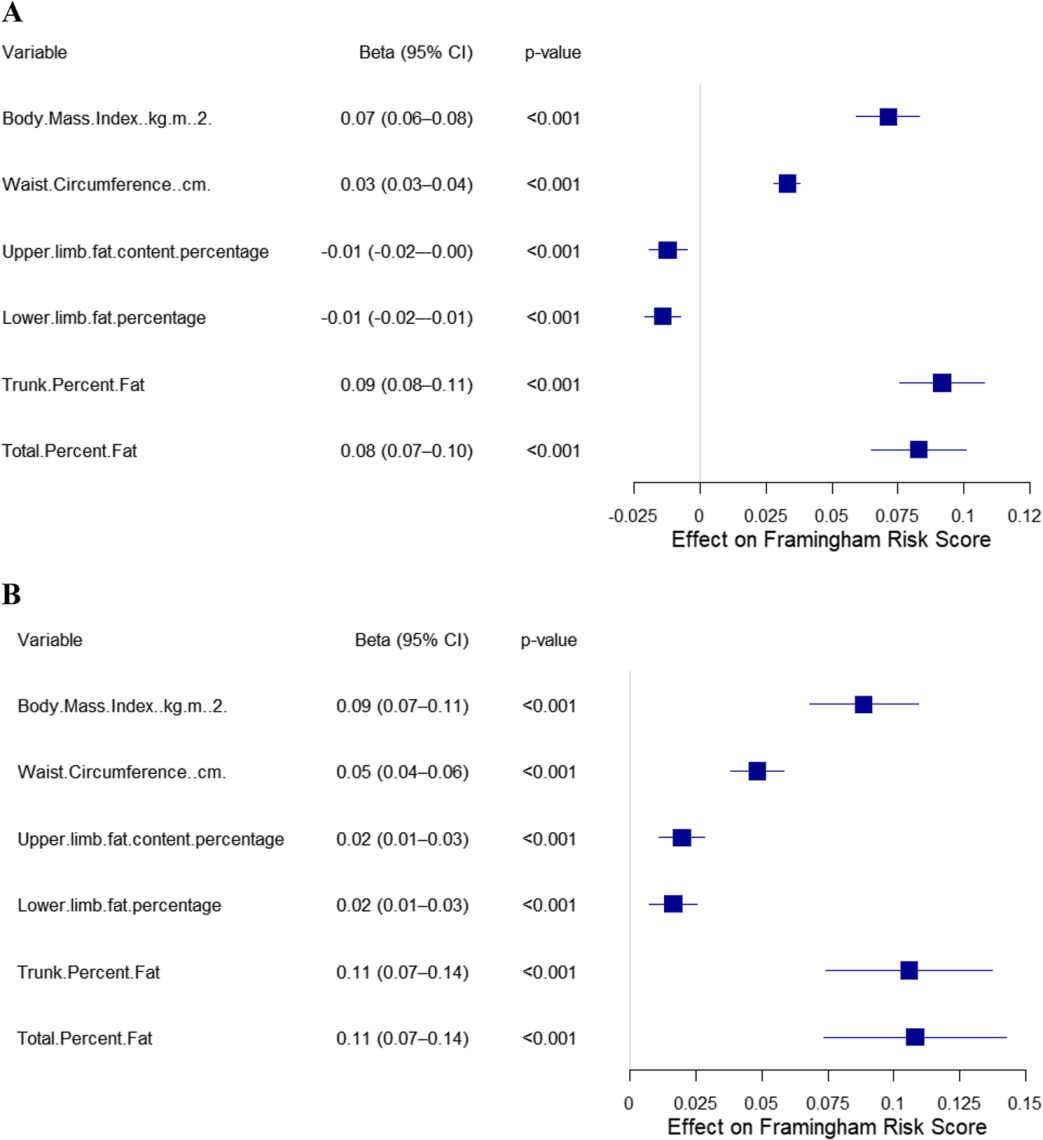
Forest Plot of the Associations Between Fat Distribution and Framingham Risk Score (FRS) in Men and WomenPanel **A** presents results for male participants; Panel **B** presents results for female participants. Note:All models were adjusted for age, race/ethnicity, education level, marital status, poverty-income ratio, smoking status, alcohol consumption, systolic blood pressure, and diastolic blood pressure. Beta coefficients represent the change in FRS per unit increase in the respective fat distribution measure.

In the fully adjusted model (Model 3), other fat distribution indicators strongly associated with FRS in women included trunk fat percentage (β = 0.11, 95% CI: 0.07–0.14, *p* < 0.001), BMI (β = 0.09, 95% CI: 0.07–0.11, *p* < 0.001), and waist circumference (β = 0.05, 95% CI: 0.04–0.06, *p* < 0.001). These results suggest that total body fat, trunk fat, BMI, and waist circumference are all important predictors of cardiovascular risk in women, with total fat percentage being the most influential. A forest plot summarizing these associations is shown in Fig. 2.

**Fig. 3:**
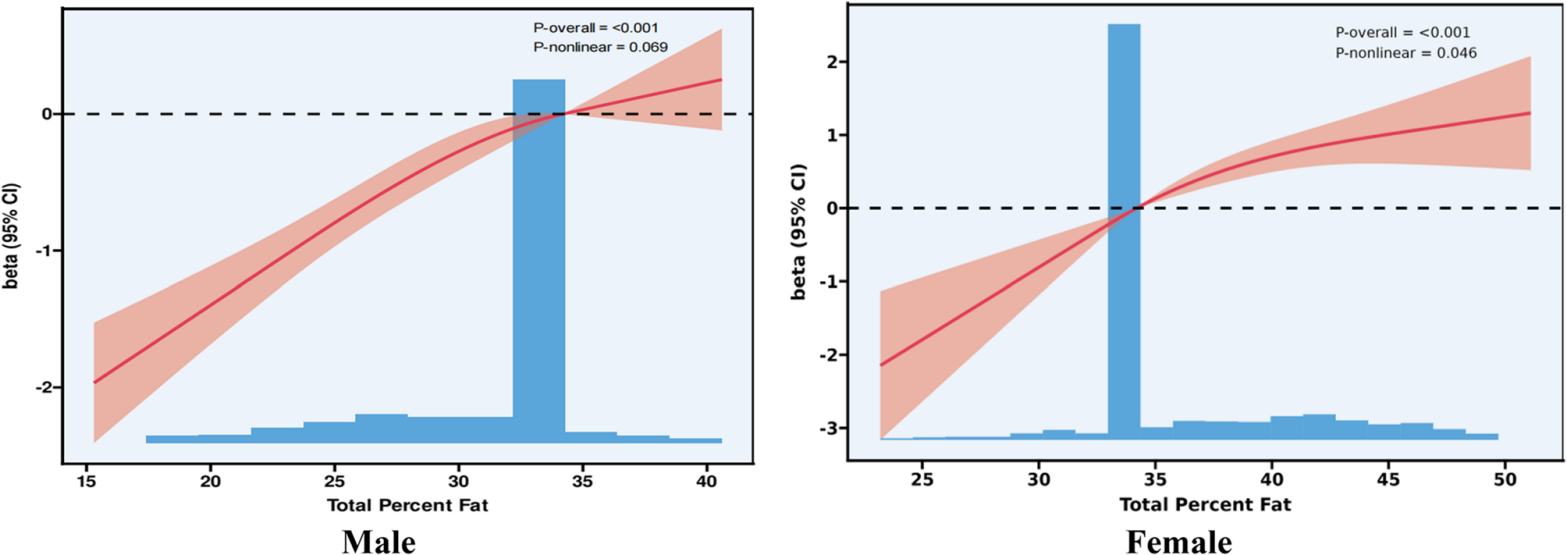
*Sex-Specific RCS Curves for Total Fat Percentage and Framingham Risk Score (FRS).Note*The left panel represents male participants and the right panel represents female participants. Red curves indicate adjusted beta coefficients with 95% confidence intervals (shaded areas), derived from multivariable linear regression models with RCS terms for total percent fat. Histograms at the bottom reflect the distribution of total percent fat in each sex. P-overall indicates the statistical significance of the association between total percent fat and FRS; P-nonlinear tests for the departure from linearity

Figure3 illustrates the restricted cubic spline (RCS) curves depicting the association between total body fat percentage and the Framingham Risk Score (FRS).Among men, a significant overall positive association was observed between total fat percentage and FRS (*P* < 0.001), although the test for nonlinearity was not statistically significant (P for nonlinearity = 0.069). The RCS curve showed a steady increase in FRS as total fat percentage rose from approximately 15–35%, indicating a linear trend in which higher adiposity corresponds to greater cardiovascular risk. Beyond 35%, the curve began to plateau slightly, suggesting a diminishing marginal effect. These findings imply that in men, total body fat is linearly and positively associated with CVD risk.

**Fig. 4:**
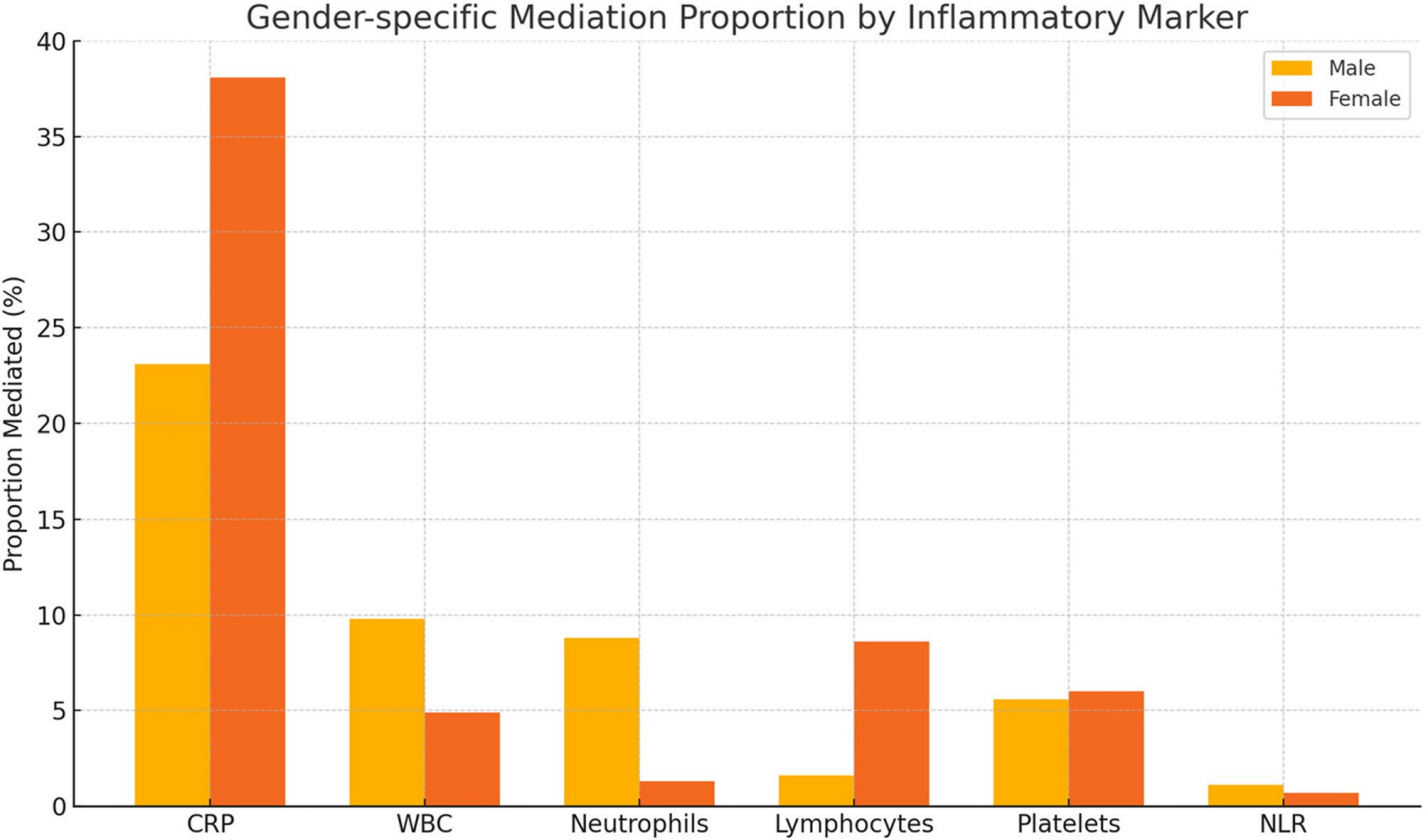
Gender-specific proportion of the total effect of total body fat percentage on Framingham Risk Score mediated by inflammatory markers. CRP accounted for the largest proportion in both sexes, especially in females (38.1%). Differences in other markers (e.g., neutrophils, lymphocytes) suggest distinct inflammatory mediation pathways between males and females.

In women, a significant nonlinear association was detected (*P* < 0.001; P for nonlinearity = 0.046). The curve revealed a U-shaped relationship: when total fat percentage was below approximately 35%, an increase in fat was associated with a decrease in FRS, indicating a possible protective effect at lower fat levels. However, beyond this threshold, FRS increased sharply with fat accumulation, suggesting an elevated cardiovascular risk. This threshold-dependent pattern highlights the importance of individualized fat management strategies in women, particularly for those with high adiposity. Figure 4

Table 3 presents the results of the mediation analysis stratified by sex.Inflammatory markers exhibited varying degrees of mediating effects in the association between total body fat percentage and Framingham Risk Score (FRS), with notable differences observed between men and women.Among men, total fat percentage exerted partial mediation through several inflammatory biomarkers. C-reactive protein (CRP) showed the strongest mediating effect, with an indirect effect of 0.01913 (95% CI: 0.01451, 0.02474) and a mediation proportion of 23.1% (95% CI: 15.9%, 31.9%; *P* < 0.001). White blood cell count (WBC) and neutrophils also demonstrated significant mediation proportions of 9.8% and 8.8%, respectively (*P* < 0.001 for both). Lymphocytes (1.6%, *P* = 0.036) and platelets (5.6%, *P* = 0.024) contributed smaller but statistically significant mediation effects. In contrast, the neutrophil-to-lymphocyte ratio (NLR) did not show a significant mediating role (1.1%, 95% CI: − 0.6%, 3.2%; *P* = 0.224). In women, CRP was also the most prominent mediator, with an indirect effect of 0.04123 (95% CI: 0.03100, 0.05393) and a mediation proportion as high as 38.1% (95% CI: 25.6%, 56.6%; *P* < 0.001), substantially greater than that observed in men. Additional significant mediators included WBC (4.9%, *P* = 0.004), lymphocytes (8.6%, *P* < 0.001), and platelets (6.0%, *P* = 0.032). However, neither neutrophils (1.3%, *P* = 0.388) nor NLR (0.7%, *P* = 0.504) demonstrated significant mediating effects in the female subgroup.

**Table 3:** *Mediation Analysis of the Associations Between Total Body Fat Percentage and Framingham Risk Score (FRS)*

Independent variable	Mediator	Total effect		Indirect effect		Direct effect		Proportion mediated, % (95% CI)
		Coefficient (95% CI)	*P* value	Coefficient (95% CI)	*P* value	Coefficient (95% CI)	*P* value	
Male								
Total.Percent.Fat	Log.CRP	0.08286 (0.06417, 0.10277)	<0.001	0.01913 (0.01451, 0.02474)	<0.001	0.06373 (0.04353, 0.08328)	<0.001	23.1 (15.9, 31.9)
Total.Percent.Fat	Log.WBC	0.08286 (0.06417, 0.10277)	<0.001	0.00813 (0.00486, 0.01180)	<0.001	0.07473 (0.05515, 0.09384)	<0.001	9.8 (5.6, 15.6)
Total.Percent.Fat	Log.Neutrophils	0.08286 (0.06417, 0.10277)	<0.001	0.00728 (0.00390, 0.01062)	<0.001	0.07558 (0.05674, 0.09547)	<0.001	8.8 (4.8, 14.2)
Total.Percent.Fat	Log.Lymphocytes	0.08286 (0.06417, 0.10277)	<0.001	0.00135 (0.00013, 0.00318)	0.036	0.08151 (0.06253, 0.10088)	<0.001	1.6 (0.2, 4.0)
Total.Percent.Fat	Log.Platelets	0.08286 (0.06417, 0.10277)	<0.001	0.00467 (0.00034, 0.00905)	0.024	0.07819 (0.05911, 0.09675)	<0.001	5.6 (0.5, 11.1)
Total.Percent.Fat	Log.NLR	0.08286 (0.06417, 0.10277)	<0.001	0.00093 (-0.00055, 0.00251)	0.224	0.08193 (0.06294, 0.10209)	<0.001	1.1 (-0.6, 3.2)
Female								
Total.Percent.Fat	Log.CRP	0.10811 (0.07494, 0.14282)	<0.001	0.04123 (0.03100, 0.05393)	<0.001	0.06687 (0.03342, 0.10181)	<0.001	38.1 (25.6, 56.6)
Total.Percent.Fat	Log.WBC	0.10811 (0.07494, 0.14282)	<0.001	0.00529 (0.00162, 0.00932)	0.004	0.10282 (0.07149, 0.13813)	<0.001	4.9 (1.6, 9.8)
Total.Percent.Fat	Log.Neutrophils	0.10811 (0.07494, 0.14282)	<0.001	0.00145 (-0.00159, 0.00491)	0.388	0.10665 (0.07477, 0.14128)	<0.001	1.3 (-1.7, 4.7)
Total.Percent.Fat	Log.Lymphocytes	0.10811 (0.07494, 0.14282)	<0.001	0.00934 (0.00505, 0.01527)	<0.001	0.09876 (0.06597, 0.13352)	<0.001	8.6 (4.3, 16.0)
Total.Percent.Fat	Log.Platelets	0.10811 (0.07494, 0.14282)	<0.001	0.00646 (0.00058, 0.01241)	0.032	0.10165 (0.06884, 0.13605)	<0.001	6.0 (0.6, 13.2)
Total.Percent.Fat	Log.NLR	0.10811 (0.07494, 0.14282)	<0.001	0.00071 (-0.00136, 0.00358)	0.504	0.10739 (0.07325, 0.14236)	<0.001	0.7 (-1.4, 4.0)

The mediation analyses were adjusted for Age in years at screening, Race/Hispanic origin, Education level - Adults 20+, Marital status, Ratio of family income to poverty, Smoking, Alcohol drinks, Systolic

Blood pres mm Hg and Diastolic

Blood pres mm Hg

## Discussion

Based on nationally representative data from the NHANES 2017–2018 cycle, this study systematically evaluated the associations between regional fat distribution and 10-year cardiovascular disease (CVD) risk, as assessed by the Framingham Risk Score (FRS). In addition, it explored the potential mediating role of inflammatory biomarkers in these associations. The findings revealed that variations in fat distribution across anatomical regions significantly influence future CVD risk, highlighting the limitations of conventional anthropometric indicators—such as body mass index (BMI) and waist circumference (WC)—in capturing the full spectrum of cardiometabolic risk.

Among men, total body fat percentage, body mass index (BMI), and waist circumference (WC) were all positively and linearly associated with the Framingham Risk Score (FRS), with cardiovascular risk increasing progressively with greater fat accumulation. Notably, total fat percentage exhibited the strongest association with FRS.These findings are consistent with prior research. For instance, one study reported a correlation coefficient of 0.328 between WC and 10-year FRS in men (*P* < 0.001), and a stronger correlation of 0.398 for BMI (*P* < 0.001) [30].

Among women, total body fat percentage exhibited a U-shaped association with the Framingham Risk Score (FRS). While moderate adiposity may confer a degree of cardiometabolic protection, cardiovascular risk increased markedly when body fat exceeded approximately 35%. This nonlinear relationship may reflect sex-specific differences in fat metabolism and distribution, although the underlying biological mechanisms remain to be fully elucidated.

One prevailing hypothesis suggests that premenopausal women preferentially accumulate fat in peripheral regions—such as the hips and lower limbs—which may exert protective metabolic effects. In contrast, postmenopausal fat redistribution toward central depots is associated with elevated risk, likely due to increased visceral adiposity. Estrogen-mediated regulation of adipose tissue expansion and inflammatory responses has been proposed as a potential contributor. However, this interpretation remains speculative, and the precise hormonal and metabolic mechanisms underlying the observed nonlinearity warrant further validation through longitudinal and mechanistic investigations [31, 32]. These findings are consistent with the fat redistribution hypothesis, which posits that the anatomical location of adipose tissue, rather than total fat mass alone, is a more accurate determinant of cardiometabolic and cardiovascular risk.

Although reducing central adiposity is generally associated with lower cardiovascular disease (CVD) risk, accumulating evidence suggests that excessive or surgically induced fat loss—such as following sleeve gastrectomy—may paradoxically exacerbate low-grade systemic inflammation due to resultant micronutrient deficiencies, including ferritin and vitamin B12 [33]. The present mediation analysis confirmed that inflammation functions as a critical intermediary in the pathway linking adiposity to CVD risk. Among the inflammatory markers evaluated, C-reactive protein (CRP) demonstrated the most robust mediating effect, accounting for 23.1% of the total effect in men and 38.1% in women. These results underscore the pivotal role of chronic low-grade inflammation as a biological mechanism underlying adiposity-related cardiovascular injury [34, 35]. Other markers—such as white blood cell (WBC) count and neutrophil count—also exhibited modest but statistically significant indirect effects. Although the neutrophil-to-lymphocyte ratio (NLR) did not reach significance in this dataset, prior studies have highlighted its prognostic value in predicting adverse cardiovascular outcomes [36]. Neutrophils contribute to inflammation through the secretion of chemotactic agents (e.g., leukotriene B4) and granule proteins such as CAMP and AZU1, which recruit monocytes. Furthermore, neutrophil extracellular trap (NET) formation activates the NLRP3 inflammasome in macrophages, promoting the release of interleukin-1β (IL-1β)—a key driver of vascular inflammation [37, 38].

The distinct temporal and functional roles of various inflammatory markers across different stages of disease progression warrant further exploration. In this study, total body fat percentage was chosen as the exposure variable in mediation models due to its strongest explanatory power for the Framingham Risk Score (FRS) in fully adjusted regressions. Nevertheless, certain regional fat indicators—such as upper and lower limb fat percentages—showed inverse associations with FRS in unadjusted models, particularly in women, suggesting potential metabolically protective roles of peripheral fat depots.Future studies should investigate whether anatomically distinct fat compartments elicit differential inflammatory responses, thereby providing a more nuanced understanding of the fat–inflammation–CVD axis.

Compared to conventional models such as the Framingham Risk Score (FRS) and polygenic risk scores (PRS), this study presents three notable advantages. First, by using DXA-based fat distribution metrics, it effectively identifies individuals with metabolically obese normal weight (MONW)—those with normal BMI but elevated central adiposity—who may be missed by traditional indicators. Second, the study goes beyond association by quantifying the mediating role of systemic inflammation in the fat–cardiovascular disease (CVD) axis, offering mechanistic insight for targeted intervention. Third, through sex-stratified analyses, it uncovers a threshold effect in women, highlighting sex-specific differences in risk trajectories and supporting tailored approaches in cardiovascular risk assessment and prevention.

This study highlights the potential cardioprotective role of lower limb fat, which showed inverse associations with cardiovascular risk in unadjusted sex-stratified models. These negatively associated fat depots merit further investigation, as prior evidence suggests that lower limb fat may act as a metabolic buffer, delaying ectopic lipid deposition in visceral tissues. Moreover, these regions are enriched in estrogen receptors and associated with elevated adiponectin levels and enhanced insulin sensitivity—mechanisms particularly relevant for premenopausal women [39, 40]. Future studies should explore the anti-inflammatory and metabolic properties of limb fat to determine whether it can serve as a protective marker in cardiovascular risk stratification [41].

From a translational perspective, these findings underscore the limitations of conventional anthropometric indices such as BMI and waist circumference, which fail to capture fat distribution heterogeneity. Incorporating high-resolution measures like DXA and accessible biomarkers such as C-reactive protein (CRP) may significantly improve risk prediction, especially among individuals with metabolically obese normal weight (MONW). A shift toward mechanism-based screening strategies that integrate fat topology and inflammatory signaling may enhance early detection and guide personalized preventive interventions.

Despite its strengths, this study has several limitations. First, the cross-sectional design restricts the ability to draw causal inferences. Although the mediation analysis provides insights into potential pathways, the causal structure of the observed associations requires validation through longitudinal follow-up studies and intervention trials [42]. Second, the validity of mediation analysis relies on several key assumptions, including the absence of unmeasured confounding between the exposure, mediator, and outcome, and the assumption that the mediator is not influenced by any confounder that also affects the outcome. Although we adjusted for a broad set of demographic and clinical covariates, the possibility of residual confounding cannot be completely excluded, and the results should be interpreted with appropriate caution.Third, the inflammatory markers assessed in this study did not include key cytokines such as interleukin-6 (IL-6) and tumor necrosis factor-alpha (TNF-α), which may offer deeper mechanistic insights. Future research could incorporate multi-omics approaches to expand our understanding of the inflammatory networks involved [43, 44]. Moreover, as the current research remains at the stage of risk identification and mechanistic hypothesis generation, future studies should focus on mechanism-driven interventions to evaluate the actual impact of body composition management and anti-inflammatory therapies on CVD risk. Practical considerations such as device accessibility, cost-effectiveness, and population acceptability must also be addressed to facilitate the broader implementation of fat–inflammation–risk assessment tools.

In conclusion, this study outlines a preliminary pathway linking fat distribution, inflammatory responses, and cardiovascular risk, and highlights both the sex-specific heterogeneity and modifiable nature of this relationship. In the context of advancing precision prevention for chronic diseases, this mechanistic model may inform the next generation of risk prediction tools, optimize clinical screening strategies, and reshape approaches to population health management.

Given the inherent limitations of cross-sectional designs in establishing causality, future studies should employ longitudinal cohorts or randomized controlled trials to validate the temporal sequence and causal mediation pathways proposed in this study. Such designs will be instrumental in confirming the fat–inflammation–CVD axis and determining whether targeted interventions—such as fat redistribution strategies or anti-inflammatory therapies—can effectively mitigate cardiovascular risk over time. In the era of digital health, the integration of Internet of Surgical Things (IoST) technologies, including wearable biosensors and remote monitoring systems, holds promise for real-time tracking of fat distribution and inflammatory biomarkers, thereby advancing individualized risk assessment and precision prevention [45].

## Data Availability

The datasets analyzed during the current study are publicly available from the NHANES website: https://www.cdc.gov/nchs/nhanes/index.htm.

## Abbreviations

CVD: Cardiovascular disease
FRS: Framingham Risk Score
BMI: Body Mass Index
WC: Waist Circumference
DXA: Dual-energy X-ray absorptiometry
CRP: C-reactive Protein
WBC: White Blood Cell
NLR: Neutrophil-to-lymphocyte Ratio
NHANES: National Health and Nutrition Examination Survey
CI: Confidence Interval
SD: Standard Deviation
